# Health-related employer support, recurring pain, and direct insurance costs for a self-insured employer

**DOI:** 10.1186/s12889-015-1784-4

**Published:** 2015-05-01

**Authors:** Jessica AR Williams

**Affiliations:** Department of Health Policy and Management, UCLA Fielding School of Public Health; (current) Harvard Center for Population and Development Studies, Harvard T.H. Chan School of Public Health, 9 Bow Street, Cambridge, MA 02138 USA

**Keywords:** Claims analysis, Health care costs, Health service research, Occupational health, Pain

## Abstract

**Background:**

Poor psychosocial workplace factors have been found to cause or exacerbate a variety of health problems, including pain. However, little work has focused on how psychosocial workplace factors, such as health-related employer support, relate to future medical expenditures after controlling for health. Health-related support has also not been well explored in previous literature as a psychosocial factor. This study estimated the association of health-related employer support and pain with future medical expenditures, after including many additional controls.

**Methods:**

This study used a restricted data set comprised of medical claims and survey data for one company in the U.S. Participants were included in the sample if they had worked for their employer for at least 12 months prior to the survey and if they were continuously eligible for health insurance (N=1,570). Future medical expenditures were measured using administrative claims data covering inpatient, outpatient, mental health and pharmaceutical insurance claims during a year. Health-related employer support was measured using participants’ answers about whether the employer would support their efforts to positively change their emotional or physical health. Pain was measured as recurring pain from any condition over the previous year.

**Results:**

Having any physical health-related employer support was associated with a 0.06 increase in the probability of having future medical expenditures greater than zero, 95% CI [0.01, 0.11], but not with total expenditures. Having pain was associated with a 0.06 increase, 95% CI [0.04, 0.09], in the probability of having future medical expenditures greater than zero and with $3,027 additional total expenditures, 95% CI [$1,077, $4,987].

**Conclusions:**

After controlling for health and pain, psychosocial workplace factors were not robustly associated with future medical expenditures. Pain was associated with increased medical expenditures for the self-insured employer in this study, adjusting for a variety of factors.

## Background

Pain is a significant problem in the U.S. During a three month period, between 15 and 29% of respondents to the National Health Interview Survey aged 18–64 reported experiencing a migraine or severe headache, neck pain, or lower back pain that lasted more than 24 hours [1]. Several studies have quantified the medical costs of certain conditions, such as musculoskeletal disorder and arthritis, and found that pain conditions are some of the most expensive health conditions–especially when the costs of reduced productivity at work were taken into account [2-8]. While pain has complex causes, physical and psychosocial workplace factors often play a role [9-13]. Job strain is one example of a psychosocial workplace factor; it denotes jobs that have high psychological demands combined with low decision authority [14]. Job strain has been associated with conditions causing pain, such as musculoskeletal conditions, as well as other health outcomes such as mortality, cardiovascular disease, and poor mental health [15-19]. Additionally, though previous studies have estimated the costs of pain to society as a whole and the costs of particular pain conditions to employers, very few have looked at employer medical costs across pain conditions while controlling for workplace factors that might also affect medical expenditures [3,5,20-24]. Using a broad rather than condition specific measure of pain allows subclinical pain to be included and allows for a broader generalization of the findings.

Physical hazards in the workplace, such as chemical exposures, have been linked to medical expenditures, primarily through Occupational Safety and Health Administration and worker’s compensation reporting systems [25,26]. In contrast, psychosocial [27,28] workplace factors have been linked more often with health than with expenditures [19,29-33]. Psychosocial workplace factors may not only affect medical expenditures though health, but may also change the opportunity cost of obtaining medical care. These effects are likely to work in opposite directions. For example, having an environment without job strain reduces the risk of cardiovascular disease but may also allow employees more leeway to go to medical appointments. One study looking at co-worker support, but not specifically for health, found evidence of a relationship between co-worker support and health care utilization [34]. Estimating the marginal effects of psychosocial workplace factors on medical expenditures will improve our understanding of their overall effects on workers and employers.

Employer support for worker efforts to change their health, an additional workplace factor that might affect medical expenditures, has not been fully explored in the literature. While most health risk reduction programs implemented by employers and vendors may exemplify some dimensions of health-related employer support, their general disconnect from working conditions, particularly from psychosocial conditions, limits their effectiveness and reach [35,36]. Working conditions are currently the target of interventions under the umbrella of Total Worker Health™ that combine individual health risk reduction with changes in work safety and organization to produce better results, as explicated by Anger et al. [35,37,38]. Without deepening our understanding of the importance of pain for employer medical costs after taking workplace conditions into account, we cannot properly estimate the potential costs and effects of interventions on pain.

Therefore, this study uses a restricted data set comprised of medical claims and survey data to estimate the associations of pain and health-related employer support with future medical expenditures after controlling for health and other factors that might independently affect future medical expenditures. Pain should be associated with increased future medical expenditures. Greater employer support for health when health, including pain, is adjusted for, should lead to a reduced opportunity cost of medical care–increasing expenditures because of greater access.

## Methods

### Data and participants

These data are from a restricted-use dataset consisting of one employer with multiple locations provided under a data use agreement with a national health total population health improvement company. The protocol for this study was approved by the UCLA Institutional Review Board (IRB#11–003195); it was not possible to obtain informed consent from each participant as the data were collected for other purposes and de-identified. The survey data came from participants in 2008. Health Insurance claims and eligibility records were obtained from the data provider from 1/1/2007 to 12/31/2009. Participants were linked in the eligibility, claims, and survey files using an encrypted identification number. Participants were included in the sample if they had worked for their employer for at least 12 months prior to the survey and if they were continuously eligible for health insurance (N=1,570).

### Measurement

Future medical expenditures were measured using administrative claims data that covered inpatient, outpatient, mental health and pharmaceutical insurance claims. The survey was given to participants in January 2008, but the exact dates were not recorded. The date of the start of the aggregation of future medical expenditures was chosen to be January 31st. Claims were aggregated for each eligible individual during the 12-month period after the survey. Prior to being aggregated, claims were adjudicated to eliminate duplicate claims. After adjudication, claims amounts that were still negative (indicating potential payment issues) were dropped from the analysis (less than 0.45% of claims). The expenditure information in the data reflects the amounts paid by the self-insured employer.

Pain was measured by participants’ answers to a three-part question about recurring pain in the past 12 months due to a neck or back condition, knee or leg condition, or other. An affirmative answer to any part was used to construct a dichotomous indicator for pain. Health-related employer support was measured using participants’ dichotomous answers to two questions on whether their employer would be supportive of their efforts to positively change their emotional or physical health. Examples of emotional health improvements were reducing stress, balancing work and home life, dealing with financial concerns, and reducing anxiety or depression. Quitting smoking and losing weight were the examples of physical health improvements. Several additional measures of psychosocial work factors were also used in the analysis: dichotomous indicators for learning or doing interesting things on the job, having fun at work, getting to use individual strengths at work, having enough resources to do the job well, job satisfaction, having a supervisor who created a trusting and open environment, and job security. The measure of whether the employee has enough resources to do their job well was dichotomized from a scale of “strongly agreed”, “agreed”, “disagreed”, or “strongly disagreed”; with agreement answers coded as one and disagreement coded as zero. The measure of job security was adapted from employee responses to a question about whether their employer was increasing, decreasing (job insecurity), or maintaining the size of its workforce–entered as a dichotomous variable indicating that the employer was not downsizing. Typical weekly hours were included as a continuous variable. The largest occupational groups were professional workers (33.10%); clerical or office workers (18.08%); manager, executive, or official workers (12.50%); and service workers (8.66%).

Physical and mental health status was measured by dichotomous indicators for asthma, high blood pressure, high cholesterol, and smoking status, self-reported body mass index (continuous), and an emotional health index (continuous). The emotional health index was based on responses to the following ten items that refer to the day before the survey: smiling or laughter, learning or doing something interesting, being treated with respect, enjoyment, happiness, worry, sadness, anger, stress, and diagnosed with depression. An additional variable, the Charlson Comorbidity Index, was derived from participants’ 2007 claims [39]. Because the sample was relatively healthy, an indicator for having a Charlson Comorbidity Index of at least one was included in lieu of the continuous scores.

Monetary resources were measured using a categorical variable for household monthly income. Household size was unavailable, so the number of children in the household under 18 and an indicator for being married or living with a partner were used to approximate household size and additional potential social support and/or increased opportunity cost of time. The type of health insurance plan was used as a proxy for out-of-pocket prices. Preferred Provider Organization (PPO), Health Maintenance Organization (HMO)-gatekeeper, HMO-Open Access, HMO–Point of Service (POS), and Indemnity were the major plan types available to participants (categorical variable). Gender and age were included in the model as measures of participants’ preferences for using healthcare.

The densities of providers and facilities were taken from the Area Health Resource File: the sum of primary care providers, specialty physicians, and psychiatrists per 10,000 people in the participant’s state and the number of hospital beds per 1,000 people in the state [40]. Indicators for the participants’ census regions were proxies for area input prices and practice patterns.

### Empirical methods

To appropriately model future medical expenditures, a distribution with many zero values and a skewed distribution, the analysis was conducted using a two-part model after applying the tests suggested in the literature. The tests included Manning and Mullahy suggestions for log transformation and a modified Park test to assess the appropriate family of distributions for the generalized linear model [41-44]. The first part was estimated using a logit model where the dependent variable was equal to one if the worker had any future medical expenditures. The second part of the model (to model the amount of expenditures conditional on having greater than zero expenditures) was a generalized linear model with gamma distribution and log link function.

Average marginal effects were calculated for each covariate for each part of the model, and for the unconditional effect (using both parts of the model). In the first part of the model the average marginal effect is the percentage point difference in the probability of having any expenditure. For categorical variables, the change in the probability of having any expenditure as the variable changes from zero to one was calculated for each observation. The estimated effects were then averaged to get the average marginal effect. For the continuous variables, the average marginal effect was the instantaneous rate of change in the probability of having any expenditure. For the second part of the model, the average marginal effect is the change in expected expenditures conditional on having expenditures greater than zero. Again, the effects were calculated for each observation and then averaged to get the average marginal effect. The unconditional effect included both parts of the model: the probability of having any expenditure multiplied by the amount of the expenditure. The average marginal effects for the complete model represent the unconditional change in expected expenditures for specified changes in the covariates. Confidence intervals were calculated using a Taylor Series expansion (STATA version 12) [45]. Missing values, including “don’t know” answers, were imputed 10 times using a multivariate normal model with categories assigned using the method described by Allison [46-48].

## Results

During the year after the survey, 7.52% of employees did not have any employer-paid medical expenditure and about 10% of the sample had expenditures double the sample mean. Nearly half, 46.00%, of the sample reported pain (see Table 1). An overwhelming majority of the sample reported having emotional health-related support (82.87%), with an even higher percentage for physical health-related support (90.06%). High levels of positive job characteristics (psychosocial factors) were reported across the board.

**Table 1:** **Unadjusted participant characteristics (N=1,570)**

**Characteristic**	**Mean (SD) or percent** ^**1**^	**Percent missing**
**Dependent variable**		
Aggregate future medical expenditures $	$8,096 ($28,522)	0.00%
**Predictors**		
Health-related employer support		
Emotional support	82.87%	1.85%
Physical support	90.06%	1.15%
Learns or does interesting things at work	83.82%	0.06%
Has fun at work	74.52%	0.32%
Has enough resources to do job well	87.71%	0.51%
Satisfied with job	93.50%	0.51%
Uses strengths every day at work	83.06%	0.13%
Supervisor creates a trusting and open environment	73.06%	8.79%
Employer not downsizing (job security)	91.21%	2.48%
Typical Hours/week	40.78 (9.81)	1.97%
Pain	46.00%	0.00%
Charlson comorbidity index score	0.45 (1.19)	0.00%
Asthma (ever told)	15.29%	0.06%
High blood pressure (ever told)	24.90%	0.06%
High cholesterol (ever told)	24.97%	0.13%
Current smoker	12.74%	0.00%
Male	41.27%	0.00%
Age (years)	45.77 (10.95)	0.00%
Body mass index	28.26 (6.37)	3.38%
Emotional health index (out of 10)	8.450 (2.26)	0.57%
Health insurance type		3.50%
PPO	64.95%	
HMO-gate keeper	18.88%	
HMO-open access or POS	12.67%	
Indemnity	3.50%	
Monthly household income		17.51%
$0 to $1,999	6.24%	
$2,000 to $2,999	8.98%	
$3,000 to $3,999	12.42%	
$4,000 to $4,999	12.23%	
$5,000 to $7,499	20.38%	
$7,500 to $9,999	9.30%	
$10,000 and over	12.93%	
Spouse or partner	74.52%	0.64%
Number of children under 18 in household	0.78 (1.07)	0.06%
Census region		0.00%
Midwest	96.43%	
Northeast	0.51%	
South	1.21%	
West	0.96%	
Health system–state level		
Hospital beds per 1,000 population	3.73 (0.67)	0.00%
Active family, General, General Internal Medicine, Pediatric, Medical Specialty Surgical Specialty, and Psychiatry physicians per 10,000 population	14.38 (1.98)	0.00%

^1^Means and standard deviations are shown for continuous variables and percentages are shown for binary and categorical variables.

The regression adjusted results and confidence intervals are displayed in Table 2. The first column displays the percentage point difference in the probability of having any expenditure (average marginal effect for the first part of the model). The second column displays the change in expected expenditures conditional on having expenditures greater than zero (average marginal effect for the second part of the model). The third column displays the unconditional change in expected expenditures (average marginal effect for both parts of the model combined). Having physical health-related employer support was associated with an increase in the probability of having any medical expenditures of six percentage points (p-value 0.03), but was not significant in the conditional part of the model or overall. None of the other psychosocial workplace factors were statistically significant at the 5% level. Having pain was associated with a six percentage point increase in the probability of having future medical expenditures (p-value <0.001). Having pain was also associated with increased expected expenditures of $2,977 conditional on having greater than zero expenditures (p-value 0.005), and with increased expenditures for the unconditional change in expected expenditures of $3,027 (p-value 0.002).

**Table 2:** **Results of the two-part model of future medical expenditures** ^**1**^

**Variable**	**Percentage point difference in the probability of having** ***any*** **expenditure** ^**2**^ **[95% CI] N=1,570**	**Change in expected expenditures conditional on** ***having > zero*** ^***2***^ **[95% CI] N=1,452**	***Unconditional*** **change in expected expenditures** ^**2**^ **[95% CI] N=1,570**
Emotional support	-0.04	$2,602	$2,256
	[-0.09, 0.01]	[-$957, $6,161]	[-$1,031, $5,544]
Physical support	*0.06*	-$2,126	-$1,748
	*[0.005, 0.11]*	[-$6,491, $2,239]	[-$5,777, $2,280]
Learns new things at work	0.02	-$1,596	-$1,386
	[-0.02, 0.06]	[-$5,051, $1,859]	[-$4,563, $1,790]
Has fun at work	-0.03	$2,401	$2,120
	[-0.06, 0.002]	[-$216, $5,017]	[-$308, $4,549]
Has enough resources to do job well	0.02	$1,231	$1,203
	[-0.03, 0.07]	[-$1,904, $4,367]	[-$1,681, $4,087]
Employer not downsizing	-0.03	$1,386	$1,193
	[-0.06, 0.01]	[-$1,666, $4,439]	[-$1,660, $4,047]
Satisfied with job	0.003	$106	$108
	[-0.07, 0.07]	[-$4,451, $4,663]	[-$4,108, $4,323]
Gets to use strengths at work	0.03	-$2,881	-$2,537
	[-0.02, 0.07]	[-$6,840, $1,078]	[-$6,166,$1,092]
Trusting and open environment created by supervisor	0.02	-$1,388	-$1,193
	[-0.02, 0.07]	[-$4,359, $1,583]	[-$3,939, $1,552]
Typical Hours Worked (hours)	-0.0004	-$13	-$13
	[-0.002, 0.001]	[-$128, $102]	[-$120, $93]
Pain	**0.06**	**$2,977**	**$3,027**
	**[0.04, 0.09]**	**[$889, $5,065]**	**[$1,077, $4,978]**
Charlson comorbidity index Score ≥1	**0.08**	**$13,080**	**$12,987**
	**[0.06, 0.10]**	**[$8,827, $17,333]**	**[$8,856, $17,117]**
Asthma	0.01	*-$2,929*	*-$2,692*
	[-0.04, 0.05]	*[-$5,154,-$704]*	*[-$4,756,-$629]*
High blood pressure	**0.05**	$389	$558
	**[0.02, 0.08]**	[-$2,096, $2,874]	[-$1,775, $2,891]
High cholesterol	*0.04*	$418	$531
	*[0.01, 0.07]*	[-$2,072, $2,908]	[-$1,799,$2,861]
Current smoker	-0.04	-$1,169	-$1,210
	[-0.08, 0.01]	[-$3,922, $1,584]	[-$3,726, $1,305]
Male	**-0.10**	**-$3,982**	**-$4,067**
	**[-0.13,-0.07]**	**[-$6,002,-$1,962]**	**[-$5,938,-$2196]**
Age (years)	0.0004	$62	$59
	[-0.001, 0.002]	[-$47, $170]	[-$42, $159]
BMI	0.001	$68	$67
	[-0.001, 0.004]	[-$103, $238]	[-$90, $225]
Emotional health index (10 point scale)	0.002	*-$566*	*-$516*
	[-0.004, 0.01]	*[-$1,072,-$60]*	*[-$984,-$48]*
Health insurance type (PPO is reference)			
HMO - gatekeeper	-0.03	-$242	-$280
	[-0.06, 0.02]	[-$2,876, $2,393]	[-$2,709, $2,150]
HMO-POS or open access	0.002	$1,170	$1,091
	[-0.05, 0.05]	[-$2,159, $4,498]	[-$1,994, $4,177]
Indemnity/HAS	-0.03	$6,160	$5,538
	[-0.11, 0.06]	[-$3,237, $15,558]	[-$3,078, $14,155]
Monthly household income (Up to $2,999 is reference)			
$3,000 to $3,999	-0.01	$1,031	$912
	[-0.06, 0.04]	[-$2,264, $4,326]	[-$2137, $3962]
$4,000 to $4,999	-0.02	$152	$65
	[-0.07, 0.03]	[-$3076, $3,381]	[-$2922, $3052]
$5,000 to $7,499	-0.02	$3,078	$2791
	[-0.06, 0.03]	[-$385, $6,541]	[-$423, $6,004]
$7,500 to $9,999	-0.004	-$804	-$758
	[-0.05, 0.04]	[-$4,185, $2,578]	[-$3,904, $2,389]
$10,000 and over	-0.01	$2,161	$1964
	[-0.06, 0.04]	[-$1,692, $6,015]	[-$1605, $5533]
Spouse/Partner	-0.001	$637	$584
	[-0.03, 0.03]	[-$1,955, $3,229]	[-$1,815, $2983]
Number of children under 18	0.01	-$975	-$878
	[-0.01, 0.02]	[-$2,067, $117]	[-$1887, $131]

^1^This regression also controlled for the number of hospital beds per 1,000 pop; Active Primary Care, Specialty and Psychiatry Specialty Physicians per 10,000 pop; and Census Region. The results are for a two-part model of future medical expenditures. The first part of the model uses a logit regression with the dependent variable equal to one if there were any expenditure. The second part of the model is a GLM (Gamma and log-link). Confidence Intervals were estimated using a first-order Taylor series expansion.

^2^Numbers in italics are statistically significant at the 5% level and that numbers in boldface are statistically significant at the 1% level.

Many of the health status covariates were strongly associated with expenditures. Having a Charlson Comorbidity Index score of at least one was associated with an increase in the probability of having medical expenditures of eight percentage points (p-value <0.001). In the conditional part of the model, having a Charlson Comorbidity Index score of at least one was associated with $13,080 of additional expenditure (p-value <0.001) conditional on having nonzero expenditure and with $12,987 greater expenditure overall (p-value <0.001). High blood pressure and high cholesterol were associated with five (p-value <0.001) and four (p-value 0.01) percentage point increases in the risk of having any expenditure respectively. Asthma was not significantly associated with the chance of having any expenditure, but was associated with reduced expenditure in the conditional part of the model and overall (p-values 0.01). Being male was associated with a decrease in the chance of having expenditure (p-value <0.001) and with reduced expenditures conditional on having positive expenditure and overall (p-values <0.001). Having a higher score on the emotional health index (indicating better emotional health) was negatively associated with the amount future medical expenditures conditional on having greater than zero expenditures, and when both parts of the model were combined (p-values 0.03).

## Discussion

There was limited evidence for the hypothesis that health-related employer support would be associated with future medical expenditures, after controlling for other factors. Physical health-related employer support was associated with increased probability of having any expenditure after controlling for pain and additional covariates, but was not significantly related to the amount of expenditures. However, none of the average marginal effects of the other psychosocial workplace measures reached conventional levels of significance. The simplest explanation for the failure of the psychosocial workplace variables to show an association with medical expenditures is that the association does not exist or that psychosocial workplace factors only affect medical expenditures through health. A weak or non-existent relationship between the workplace psychosocial factors used in this study *after* controlling for health, suggests that better psychosocial conditions might not increase medical costs for employers. Another possibility is that the sample size was too small or lacked enough variation (the workforce was largely professional) in psychosocial workplace factors to accurately determine the nature of the relationship. Regardless, it does not seem that having positive psychosocial workplace factors strongly increases healthcare utilization when health is included in the model.

Pain contributes to greater medical expenditures even after controlling for several measures of health status and the psychosocial workplace factors, as expected. The estimate from this study that employees with pain had an additional $3,027 (95% CI [$1,077, $4,978]), in medical expenditures compared to individuals without pain is lower than the estimates of Gaskin and Richard, who estimated that individuals with pain had an additional $4,516 of expenditures compared to those without pain [2,20]. However, Gaskin and Richard used a nationally representative sample–which is very different from the employee population used in this sample [2,20]. While the prevalence of pain was smaller in the national sample (21% versus 46% in this study) individuals with severe functional limitations from pain are less likely to be employed and are more likely to have greater expenditures because of the severity of their pain [2,20]. Additionally, the SF-12 (12-Item Short-Form Health Survey), which measures bodily pain during the *previous week* was used in the national sample whereas the measure of pain used in this study covered the *previous 12 months* [2,20].The results also showed a negative association between emotional health and future healthcare expenditures, which highlights the possibility for emotional health to be an additional target for workplace health interventions.

### Limitations & generalizability

The major limitations of this study were the relatively small sample size, having a single employer, and the limited duration of follow-up. Even though participants worked at different sites, there was little variation in their answers to questions about the psychosocial work environment. There may also have been unobserved characteristics of the participants, such as personality characteristics, that affected their perceptions of health-related employer support, pain, and future medical expenditures. Future studies would be improved by including proxies for these characteristics. Additionally, the survey and most of the time period used for claims analysis occurred during an economic downturn that lasted from June 2007 to June 2009 [49]. The additional uncertainty of the time may have affected participants’ views of their employer and job characteristics.

## Conclusions

After controlling for health, the psychosocial workplace measures used in these analyses were not robustly associated with future medical expenditures, although there was suggestive evidence that having health-related employer support was associated with a greater probability of having any expenditures. Pain was strongly related to great future medical expenditures even after controlling for a variety of other factors. Better emotional health was linked to lower expenditures. Future work using larger samples and additional psychosocial workplace measures, such as Job Strain and Effort-Reward Imbalance might be able to more clearly identify whether there is a relationship between psychosocial workplace measures and medical expenditures above and beyond the effect of psychosocial workplace measures on health. Additionally, pain and emotional health might be rewarding targets for workplace interventions to improve worker health and reduce medical costs for employers.
